# Linking individual phenotype to density-dependent population growth: the influence of body size on the population dynamics of malaria vectors

**DOI:** 10.1098/rspb.2011.0153

**Published:** 2011-03-09

**Authors:** Tanya L. Russell, Dickson W. Lwetoijera, Bart G. J. Knols, Willem Takken, Gerry F. Killeen, Heather M. Ferguson

**Affiliations:** 1 Biomedical and Environmental Thematic Group, Ifakara Health Institute, PO Box 53, Ifakara, Tanzania; 2 Department of Biological and Biomedical Sciences, University of Durham, South Road, Durham DH1 3LE, UK; 3 Vector Group, Liverpool School of Tropical Medicine, Pembroke Place, Liverpool L3 5QA, UK; 4 School of Population Health, Australian Centre for Tropical and International Health, The University of Queensland, Brisbane 4006, Australia; 5 Department of Zoology and Marine Biology, University of Dar es Salaam, PO Box 35064, Dar es Salaam, Tanzania; 6 Division of Infectious Diseases, Tropical Medicine and AIDS Academic Medical Center, F4-217, Meibergdreef 9, 1105, Amsterdam, The Netherlands; 7 Laboratory of Entomology, Wageningen University and Research Centre, PO Box 8031, 6700, Wageningen, The Netherlands; 8 Institute of Biodiversity, Animal Health and Comparative Medicine, University of Glasgow, Glasgow G12 8QQ, UK

**Keywords:** density dependence, body size, phenotypic plasticity, population dynamics, malaria, *Anopheles gambiae*

## Abstract

Understanding the endogenous factors that drive the population dynamics of malaria mosquitoes will facilitate more accurate predictions about vector control effectiveness and our ability to destabilize the growth of either low- or high-density insect populations. We assessed whether variation in phenotypic traits predict the dynamics of *Anopheles gambiae sensu lato* mosquitoes, the most important vectors of human malaria. *Anopheles gambiae* dynamics were monitored over a six-month period of seasonal growth and decline. The population exhibited density-dependent feedback, with the carrying capacity being modified by rainfall (97% *w*AIC_c_ support). The individual phenotypic expression of the maternal (*p* = 0.0001) and current (*p* = 0.040) body size positively influenced population growth. Our field-based evidence uniquely demonstrates that individual fitness can have population-level impacts and, furthermore, can mitigate the impact of exogenous drivers (e.g. rainfall) in species whose reproduction depends upon it. Once frontline interventions have suppressed mosquito densities, attempts to eliminate malaria with supplementary vector control tools may be attenuated by increased population growth and individual fitness.

## 1. Introduction

Recent evidence suggests the population dynamics of most taxa are influenced by both endogenous (density-dependent) and exogenous (density-independent) processes [1,2]. This challenges the concept that the population dynamics of fast-growing insect species are solely governed by exogenous processes, such as climate, water and resource availability [3]. The assumption that endogenous factors play a negligible role on population dynamics has influenced many of the conceptual and practical approaches used for controlling insect pest and vector species. Ignoring endogenous processes could lead to exaggerated predictions about effectiveness by implying that control techniques will be equally effective at destabilizing the growth of low- and high-density insect populations [4]. If population growth is under strong endogenous regulation, control measures may become proportionately less effective at lower densities because remaining individuals will compensate with enhanced reproduction and survival [4,5]. Studies of economically and medically important insect vectors frequently demonstrate that the phenotypic traits that mediate individual fitness (e.g. body size) are optimized as population density falls [6–9]. If such individual-level effects can scale up to influence population growth, it is possible that the impact of interventions targeted solely at abundance will become weakened as the density of pest populations is reduced. Understanding the concurrent roles of exogenous and endogenous factors on population growth is thus crucial for predicting the response of vector populations to density-reduction strategies, and is of widespread interest to population ecologists and programme managers.

As the international community has now prioritized national and regional malaria elimination, with a long-term ultimate goal of eradication [10], the need to accurately predict mosquito vector population responses to perturbation is very timely. The aim of the current study was to investigate this issue by estimating the contribution of exogenous environmental factors and endogenous demographic and phenotypic traits to the seasonal population dynamics of mosquitoes in the *Anopheles gambiae* species complex *sensu lato* (s.l.). *Anopheles gambiae* s.l. are the primary malaria vectors in sub-Saharan Africa and are responsible for 500 million clinical cases annually, from which 1 million people die [11]; but this disease burden is dropping across most of Africa, where vector control with insecticide-treated nets (ITN) or indoor residual spraying is practised (e.g. [12,13]). For malaria control and elimination there is an urgent need not only to develop new control tools [10,14], but also to understand how to suppress the growth of vector populations across a range of ecological settings [15].

The dependence of *A. gambiae* s.l. population dynamics on exogenous environmental variables such as rainfall and temperature has been well documented [16–18]. The strong dependence on rainfall occurs because larval mosquitoes require aquatic habitats (typically small, ephemeral freshwater pools), and temperature regulates their development within these habitats [19]. Perhaps on account of these clear environmental influences on *A. gambiae* s.l. dynamics, the additional impacts of endogenous factors are often overlooked and have rarely been investigated under natural conditions. However, the application of advanced statistical models to mosquito time-series data have recently revealed a strong role of endogenous factors in regulating the dynamics of other tropical mosquitoes [20,21]. These studies highlight the need to simultaneously consider both exo- and endogenous drivers when predicting mosquito population dynamics, as their interaction may be more important than either factor on its own.

The hypothesis that density-dependent factors and phenotypic traits could similarly impact *A. gambiae* s.l. dynamics is drawn from the observation that several adult fitness and life-history traits are known to be influenced by larval density under laboratory conditions. Numerous studies have demonstrated that larval density is negatively correlated with individual adult fitness traits including body size [7–9], energetic reserves [22,23], survival [22,24,25], female blood intake, egg production [26] and male mating competitiveness [25]. Of these, adult body size is generally the best predictor of adult fitness because of its positive correlation with female fecundity and adult survival, in these mosquitoes and many other insects [6]. However, the effect of plasticity in adult body size as an endogenous driver of population dynamics of insects, including mosquitoes, has not been rigorously quantified, even though individual fitness has been demonstrated to scale up to influence dynamics of other taxa [27,28].

Here, we tested whether variation in the mean body size of current and maternal generations could be a significant endogenous driver of *A. gambiae* s.l. dynamics under natural conditions. In seeking to identify the role of phenotypic plasticity in body size, we aim both to provide a more realistic understanding of the sources of variation in mosquito population dynamics, and to enable better predictions about their likely responses to vector control interventions.

## 2. Methods

### (a) Study area

The study was conducted in the neighbouring villages of Namawala and Idete, located in the Kilombero Valley (8.1° S, 36.6° E), southeastern Tanzania (fig. 1 in [29]). These communities experience hyper-endemic malaria transmission, primarily vectored by large populations of *A. gambiae* s.l. In this area, this species complex is represented by the two morphologically identical sibling species: *A. gambiae sensu stricto* (s.s.) Giles and *Anopheles arabiensis* Patton. Annual rains (December–May) create large quantities of ephemeral aquatic habitat suitable for *A. gambiae* s.l. oviposition and larval development.

**Figure 1.**
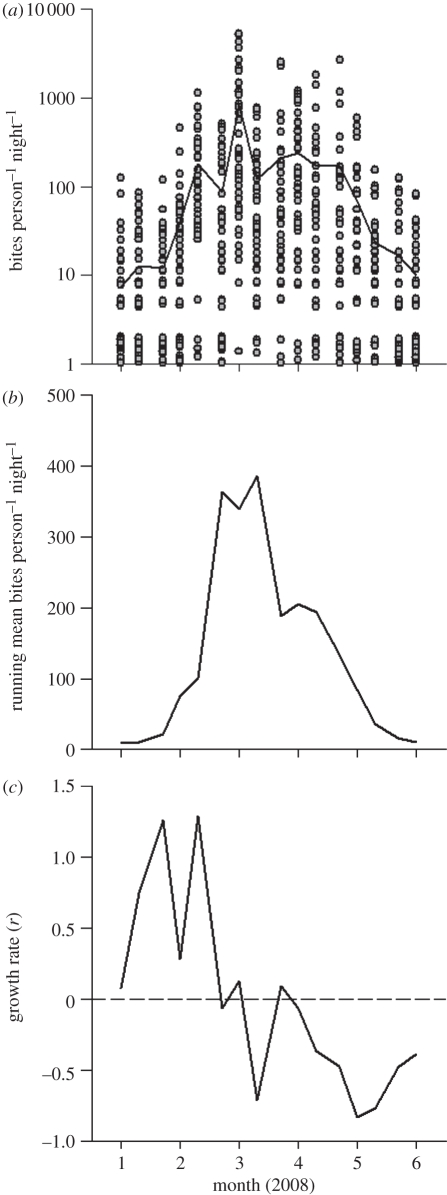
Weekly (*a*) crude (circles: raw data; solid line: arithmetic mean), (*b*) running density and (*c*) *per capita* population growth rate of *A. gambiae* s.l. females.

### (b) Population sampling

Seventy-two households were randomly selected for mosquito sampling in both villages using census information from the Ifakara Health Institute (IHI) Demographic Surveillance System. If selected households were unwilling to participate in the study, additional households were randomly selected until the full quota was reached. Houses were dispersed over approximately 50 km^2^ in each village [29]. The mosquitoes were sampled from interbreeding, genetically homogeneous populations of both *A. gambiae* s.s. and *A. arabiensis* [30].

Each house was visited once a month (six houses per day, four days per week for three weeks per month) over a period of six months (January–June 2008). Mosquitoes were collected inside houses using one light-trap placed beside a person sleeping underneath an ITN (Olyset, A to Z Textile Mills, Tanzania) and left to run for 12 h (19.00–07.00) [31]. The light-trap, fitted with an incandescent bulb, was placed 1–1.5 m above the floor and close to the feet of the ITN occupant. Light-traps capture host-seeking female mosquitoes as a proxy measurement of the human-biting rate [31]. Traps were inspected each morning and all mosquitoes that were morphologically identified as *A. gambiae* s.l. were visually classified into sex and feeding status groups (females: unfed, partially fed, fully fed or gravid [32]).

### (c) Endogenous measurements

Owing to the large number of female mosquitoes caught per trapping effort (up to approx. 1500), separate random sub-samples, each averaging approximately 10 per cent of the total in each trap, were used to provide an estimate of the mean female body size of the population. In cases where the catch was less than 10 females, measurements were taken from all individuals. The phenotypic trait of body size was estimated from the proxy of wing length [26], which is known to be positively correlated with body size in many insects, including mosquitoes [26,33]. Wing length measurements were made using a dissecting microscope (Nikon SMZ645) with an ocular micrometer scale (1 unit = 0.35 mm). Each sub-sampled mosquito also underwent molecular analysis to identify species (*A. gambiae* s.s. or *A. arabiensis*) by polymerase chain reaction (PCR) [34]. Prior to molecular analysis, mosquitoes were stored at −20°C in micro-centrifuge tubes containing a small amount of silica drying agent.

### (d) Exogenous environmental measurements

Weekly data on rainfall throughout the study period were obtained from the nearby Kilombero Agricultural Training and Research Institute (less than 12 km from Idete village). Measurements of temperature and relative humidity (RH) were obtained from data loggers (Tinytag TV-1500, Gemini Data Logger, UK) installed as part of long-term climatic surveillance in nearby Lupiro village. Data loggers recorded the parameters hourly from inside four representative sentinel houses to estimate variation in the daily microclimatic conditions experienced by resting mosquitoes.

### (e) Mosquito population dynamics

Time-series of the average abundance and wing length of female *A. gambiae* s.l. were calculated from the 48 houses sampled each week in both villages, over the six-month study period. Prior to analysis, the raw weekly mean mosquito abundance was smoothed using a centred moving average (calculated as an unweighted mean from three adjacent weeks) to minimize short-term variations from the long-term trend. Variation in mosquito abundance between sampling points was used to calculate the *per capita* population growth rate (*r*_*t*_ = log_e_ [*N*_*t*+1_/*N*_*t*_], where *N*_*t*_ = population density at time *t*, and sampling points were one week apart).

After computing the *per capita* growth rate through time, a series of concurrent statistical models were fitted to test its association with both contemporary and time-lagged exo- and endogenous covariate factors. A two-week time-lag period was chosen to provide a measure of the environmental conditions and phenotypic characteristics of the maternal generation of the population whose growth was under consideration. Under typical field conditions, it takes 5–12 days for eggs to develop into larvae and then pupae [7], with the resulting adults producing a new generation of eggs approximately 4 days later [35,36]. Consequently, the minimum and maximum period between generations is in the range of 9–16 days, and is reasonably approximated as two weeks.

All models were fitted using maximum-likelihood mixed-effects linear regression (generalized linear mixed model, GLMM) in the R statistical software package (v. 9.2.1) using the lmer function [37]. The randomized allocation of households resulted in a different cluster of households being sampled each week, with each cluster being repeatedly sampled on a monthly scale. As such, the models included a random variable for cluster to account for this repeated sampling. The data from both villages were pooled to analyse demographic growth at the population level. This was justified because the distance between houses/villages in relation to average dispersal distances of these mosquitoes was small [38] and, crucially, there was a lack of any genetic structuring between mosquitoes in these sites, as confirmed by concurrent population genetic analyses [30]. Preliminary statistical analysis indicated that there was no significant difference in the *per capita* growth rates observed for each village (*p* = 0.5317; GLMM, explanatory variables incorporated both density and village).

Highly correlated or interacting factors can reduce the strength of conclusions based on their main effects. Therefore, the presence of correlations and interactions between the exo- and endogenous factors were evaluated. Correlations between factors were tested using Pearson correlations. Interactions between each of the factors, including density, were investigated using full-factorial GLMMs. Any factors that interacted directly with density were considered to have the ability to influence the environmental carrying capacity.

The strength of evidence for density-dependent feedback on *per capita* growth rate was then investigated. Competing statistical models were fitted to the observed data to evaluate the most likely model of *A. gambiae* s.l. population dynamics over the study period. The models were selected *a priori* from a common set of density-independent and density-dependent models, which have emerged from theoretical studies of population dynamics (see [1,2]). When fitting the following models, all parameters were computed using the observed data, unless specified. First, statistical support was investigated for two density-independent models. The most basic model assumed random population growth with stochastic fluctuations around a mean rate of zero. This process is referred to as ‘random walk’ where the maximal intrinsic growth rate is equivalent to zero and the population is at equilibrium:2.1
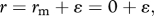
where *r*_m_ = maximal intrinsic observed *r*, and *ɛ* = an error term with a mean of zero and a variance of *σ*^2^. A second density-independent model was tested that assumed population growth was exponential, with the carrying capacity (*K*) being unlimited (*K* = ∞):2.2
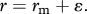


For the density-dependent models, we investigated alternative dynamics by using either a fixed or variable carrying capacity. Density-dependent models modify the rate of population growth (*r*) in relation to the proximity of the current population density (*N*) to the carrying capacity (*K*), and are conventionally modelled by variants of the theta-logistic equation [1,2]:2.3
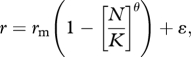
where *r*_*m*_ is as before, *N* is the current observed density, *K* being equivalent to the maximum observed density and *θ* a parameter describing the curvature of the relationship. The simplest model of density dependence investigated assumed a linear relationship between population growth rate and density (*θ* = 1), defined as the Ricker-logistic model. Next the Gompertz-logistic model was investigated, which assumes a negative log-linear relationship between population growth and density:2.4
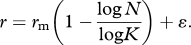


This model predicts that values of *r* are highest at low population densities, but that growth quickly reduces as density increases and eventually reaches an asymptote.

The above variants of the theta-logistic model assume that the carrying capacity of the population is fixed, but this is unlikely for species living in fluctuating environments where the availability of resources critical for reproduction and survival vary substantially through time [39,40]. Rainfall—the major driver of larval habitat production [17,18]—was the only factor that interacted with density (see §3), and thus is likely to alter the carrying capacity of the population. To investigate this possibility, we fitted variants of the Ricker-logistic (equation (2.5)) and Gompertz-logistic (equation (2.6)) models that modified the carrying capacity in relation to concurrent rainfall:2.5
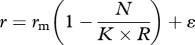
and2.6
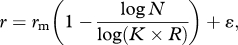
where *R* is the total weekly rainfall. To ensure that rainfall was not able to explain more variation in *r* as an independent factor (rather than as a modifier of *K*), it was also independently added to each of the density-independent and density-dependent models.

Quantitative multimodel inference (MMI) selection procedures were applied to select which models best described *A. gambiae* s.l. population growth rate through time. Model selection was based on ranking the value of the Akaike's information criterion corrected for small sample sizes (AIC_c_) [41] computed for each of the alternative models. Although the AIC_c_ values themselves are not informative [41], their relative differences can be used to select the model with the highest degree of statistical support. The difference between each AIC_c_ value relative to the model with lowest value (AIC_min_) was computed for all *i* alternative models as Δ_*i*_ = AIC_*i*_ − AIC_min_ (with Δ_*i*_ = 0 for the most likely model; the remaining models have positive values and models with Δ_*i*_ ≤ 2 considered to have substantial support). The relative strength of evidence for each model within the set of alternatives was assessed using Akaike weights (*w*AIC_c_), calculated as:2.7
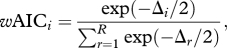
where the *w*AIC_c_ for each model is interpreted as the probability that model *i* is the most likely model within the entire set of models, with support varying from 0 (no support) to 1 (total support) [41–43].

Once the most likely base model for mosquito population dynamics was identified from the candidates described above, the role of additional contemporary and maternal-lagged exo- and endogenous factors were investigated. A total of six additional explanatory variables were considered including contemporary and maternal-lagged measures of maximum temperature, minimum humidity and mean wing length. The factors of maximum temperature and minimum humidity were used because they were considered to be more biologically limiting than the mean or alternative extreme [16,17,19].

The influence of these additional exo- and endogenous factors on population growth rates was investigated by adding them to the base model in a step-forward and step-backward fashion. Each explanatory factor was tested individually and ranked using MMI to assess which factor had the most significant impact. The most likely models from each selection step formed a series of nested models. The most parsimonious model from the final set of nested models was compared with the likelihood ratio test (2 log(*L*_2_/*L*_1_) = 2[log(*L*_2_) − log(*L*_1_)]) and compared with the *χ*^2^ distribution [44]. To ensure that the final model predicted realistic growth rates, a simulated population was constructed using a logical grid of all model factors to predict the growth rates. The differences between the simulated and observed populations were compared using multivariate analysis of variance.

### (f) Ethics

Ethical approval for the study was obtained from the IHI Institutional Review Board (IHRDC/IRB/No. A-32) and the Medical Research Coordination Committee of the National Institute for Medical Research (NIMR/HQ/R.8a/Vol. IX/764) in Tanzania. When the study commenced, permission was obtained from each household owner, who, after consenting, signed an informed consent form stating their willingness to participate in the study.

## 3. Results

During the six month sampling period, a total of 804 light-trap nights of sampling were conducted. A total of 84 030 female mosquitoes were caught, of which 98.5 per cent were unfed, and thus considered to have been host-seeking. Of these mosquitoes, 35.4 per cent (*n* = 29 775) were *A. gambiae* s.l., comprising 86.1 per cent *A. arabiensis* and 13.9 per cent *A. gambiae* s.s. (*n* = 3865 PCR amplifications). The average number of *A. gambiae* s.l. per light-trap was 37.0 ± 4.5. The remaining mosquitoes were 1.3 per cent *A. funestus* (*n* = 1119), 58.7 per cent *Culex* spp. (*n* = 49 303), 2.0 per cent *Mansonia* spp. (*n* = 1695) and 2.5 per cent other species, including *Aedes* and *Coquillettidia* spp. (*n* = 2605).

**Figure 2.**
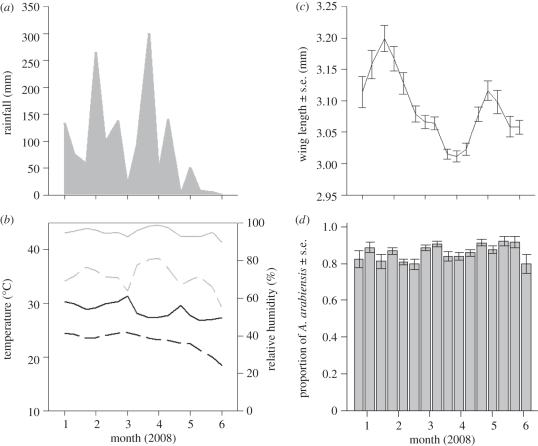
Weekly variation of exogenous factors of the study site (*a*) rainfall, and (*b*) temperature (solid black line, maximum; dashed black line, minimum) and humidity (solid grey line, maximum; dashed grey line, minimum); and endogenous factors of the *A. gambiae* s.l. population (*c*) wing length and (*d*) proportion of *A. arabiensis*. Regarding species composition in (*d*), the *A. gambiae* s.l. population (represented as 1.0 on the *y*-axis scale) was composed only of *A. arabiensis* and *A. gambiae* s.s.

Over the course of the study period, the population growth rate (*r*) of *A. gambiae* s.l. varied substantially and fluctuated between +1.3 and −0.9 *per capita* growth per week. As expected, mostly positive growth was observed at the start of the rainy season when larval habitat increased, and mostly negative growth occurred at the end of the rainy season (figure 1). Rainfall varied extensively but remained relatively high during the first four months of the study (January–April; figure 2*a*), with mosquito population density peaking in March. The maximum temperature and minimum humidity were highest during the first month of the study (January) and then declined with time (figure 2*b*).

The average female wing length varied between 3.01 ± 0.001 and 3.19 ± 0.019 mm throughout the study, with the mean being largest around January and February, after the commencement of the wet season (figure 2*c*). The mean wing length of *A. gambiae* s.l. was 3.087 ± 0.004 mm (range = 1.88–3.88). Consistent with published literature [45], the mean wing length of *A. arabiensis* (3.085 ± 0.005 mm, range = 2.25–3.75) was larger than its sibling species *A. gambiae* s.s. (3.046 ± 0.012 mm, range = 1.88–3.63; GLMM: *χ*^2^ = 9.44, d.f. = 4, *p* = 0.002). The ratio of *A. arabiensis* to *A. gambiae* s.s. remained constant throughout the study (binomial GLMM with a categorical explanatory variable for week and adjusted for multiple comparisons with Dunnett's test; *p* > 0.05; figure 2*d*), thus temporal variation in mean body size is unlikely to be owing to changes in species composition. Therefore, the population models presented here were fitted to the species complex; furthermore, similar population dynamics were observed when the models were fitted to each sibling species.

**Figure 3.**
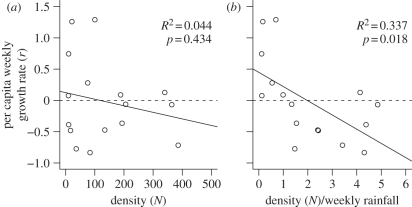
The relationship between *A. gambiae* s.l. population growth rate and (*a*) current density and (*b*) current density scaled against weekly rainfall (relative estimate of carrying capacity).

The exo- or endogenous factors were not correlated (see electronic supplementary material, table SA1), therefore all factors were included in the final analysis. There were no interactions between each of the factors (*p* > 0.05) with the exception of rainfall, which interacted with density (*p* = 0.003), indicating that the effect of density on *per capita* growth depended on rainfall levels. Low population densities (i.e. *n* < 100) were accompanied by both low and high population growth rate values (*r*), depending on the time of sampling (figure 3*a*). This reflects the biological reality of how the carrying capacity (*K*) of the population was modified by seasonal changes in rainfall (figure 3*b*). At the start of the rainy season mosquito density was low, but as the number of larval habitats begun to increase the population exhibited rapid growth. Similarly, low population densities were observed at the end of the rainy season when the quantity of larval habitat was substantially reduced and becoming limiting, and were correlated with negative instead of positive growth.

When investigating the strength of density-dependent feedback on *r*, there was little support for the density-independent models (random walk and exponential growth), density dependence assuming a fixed carrying capacity (Ricker-logistic and Gompertz-logistic) or the addition of rainfall as an independent factor to these density-independent and density-dependent models (table 1). Of the two models that modified the carrying capacity by rainfall, there was little support for the rainfall-modified Gompertz-logistic model. The most likely model (97% *w*AIC_c_ support) of *A. gambiae* s.l. population dynamics incorporated a fluctuating carrying capacity linked to rainfall based on the Ricker-logistic density-dependent model (table 1).

**Table 1.** Alternative statistical models evaluated to determine the baseline population dynamics the *Anopheles gambiae* s.l. Model comparison was made using ΔAIC_c_ and *w*AIC_c_.

model	d.f.	log likelihood	ΔAIC_c_	*w*AIC_c_
random walk	3	−16.36	9.9297	0.0068
exponential	3	−16.36	9.9297	0.0068
Ricker-logistic	4	−16.50	13.8438	0.0009
Gompertz-logistic	4	−16.13	13.1069	0.0013
random walk + rainfall	4	−18.68	18.2227	0.0001
exponential + rainfall	4	−18.68	18.2227	0.0001
Ricker-logistic + rainfall	5	−17.45	20.1251	<0.0001
Gompertz-logistic + rainfall	5	−16.04	17.2941	0.0001
rainfall-modified Ricker-logistic	4	−9.57	0.0000	0.9754
rainfall-modified Gompertz-logistic	4	−14.36	9.5801	0.0081

**Figure 4.**
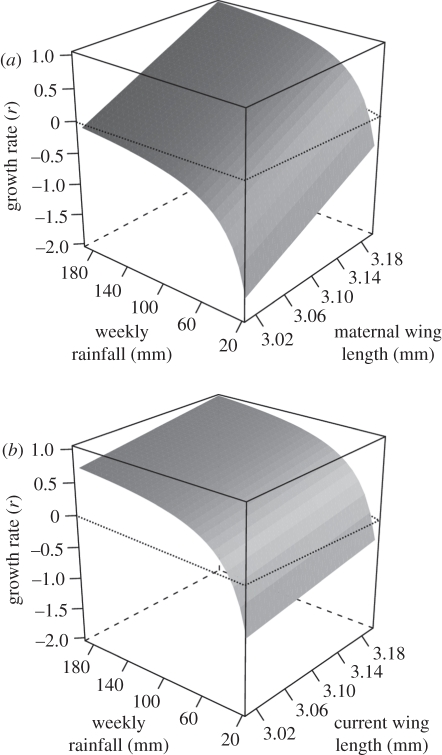
The predicted influence of rainfall as well as (*a*) maternal and (*b*) current wing length on the population growth rate of the *A. gambiae* s.l. as obtained from the best-fit statistical model of their dynamics over a six-month seasonal period of expansion and decline. The surface shows the predicted growth rate (*r*) based on rainfall-modified Gompertz-logistic density dependence and maternal or current wing length.

**Figure 5.**
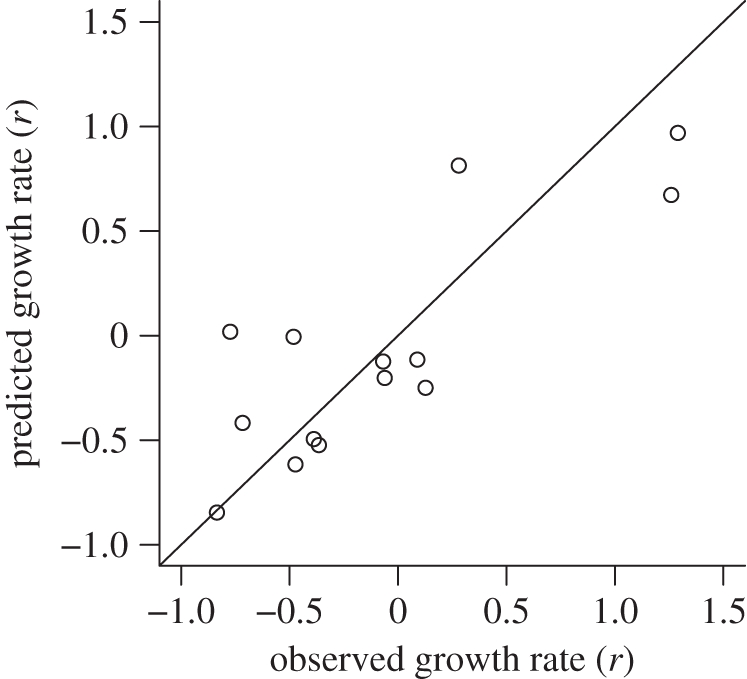
Comparison of the observed and predicted growth rates (*r*). The predicted growth rates were simulated using the rainfall-modified Ricker-logistic density-dependent growth model incorporating factors for mean maternal and current wing length.

The rainfall-modified Ricker-logistic model was therefore selected as the base model for evaluating the influences of additional exo- and endogenous factors on population dynamics. Both the step-forward and step-backward models' selection procedures supported the same final model; as such only the results of the step-forward procedure are presented, for simplicity. In the first round of model selection, the base model was most substantially improved by adding the mean maternal wing length (58% *w*AIC_c_ support; electronic supplementary material, table SA2). After the addition of maternal wing length, there was 33 per cent *w*AIC_c_ support for adding the mean current wing length (see electronic supplementary material, table SA2). The addition of both the maternal and current wing length was significant when the nested models were compared with the log-likelihood ratio test (table 2). None of the other remaining candidate factors were able to further improve model fit (see electronic supplementary material, table SA2). As such, the model that best predicts population growth of *A. gambiae* s.l. mosquitoes through the seasonal cycle of population expansion and decline includes ambient rainfall as well as mean maternal and current wing length (figure 4). The additional environmental factors of maximum temperature and minimum humidity (figure 2*b*) were unable to add any further significant explanatory power. There was no significant difference between the population simulated with this model and the observed values (*F* = 1.199, d.f. = 1, *p* = 0.308; figure 5).

**Table 2.** Series of nested models evaluated to determine which best predicted the population dynamics of *Anopheles gambiae* s.l. Model comparison was made on the basis of ΔAIC_c_, *w*AIC_c_ and goodness-of-fit using maximum-likelihood estimation.

model	d.f.	log likelihood	AIC_c_	ΔAIC_c_	*w*AIC_c_	test d.f.	*χ*^2^	*p*-value
rainfall-modified Ricker-logistic	4	−9.57	30.786	9.68	0.005			
rainfall-modified Ricker-logistic + maternal wing length	5	−2.55	21.110	0	0.633	1	11.62	0.0001
rainfall-modified Ricker-logistic + maternal wing length + wing length	6	−0.45	22.233	1.12	0.361	1	0.07	0.040

Population growth was maximized when both rainfall and the average maternal and current body size were highest. The rapid population expansion observed during January–February (figure 1) was most probably facilitated by the occurrence of large (and highly fecund) individuals at relatively low densities, since rainfall commenced in December. Notably, by April the density and growth of the population had begun to decline even though larval habitat was still abundant, with rainfall levels remaining high. During this time, the average maternal and current wing length became smaller, suggesting that mosquitoes had begun to experience competition for larval resources within aquatic habitats. Consequently, further positive population growth was limited by endogenous feedback from the parental and current generations. The factors of rainfall and wing length were not correlated with one another (see electronic supplementary material, table SA1), suggesting that their effects on population growth rate are mediated through independent interactions with the environment, with rainfall having a disproportionate effect on increasing low-density populations and competition-mediated reductions in body size primarily limiting high-density populations.

## 4. Discussion

Elucidating the mechanisms that drive the population dynamics of insect pest species is vital for predicting future population dynamics and using this knowledge to destabilize population growth. Although it has been recognized that the population dynamics of most taxa are influenced by both exogenous and endogenous processes [1,2,39], the influence of endogenous factors on mosquito populations are often overlooked, perhaps on account of clear exogenous influences such as climate and water [3]. Here, we present results from a natural mosquito vector population that show this species exhibits both direct and delayed density-dependent population growth. The direct density dependence arose from negative feedback on growth rates mediated by their contemporary larval population density and body size. This finding supports the emerging consensus that density dependence is ubiquitous for a range of taxa, including invertebrates, mammals, birds and fish [1,2]. The delayed density dependence was implicated from changes in a phenotypic trait of the maternal generations, which predicts the population dynamics of the offspring cohort. Clearly, the population dynamics of this important malaria vector are more complicated in reality than is commonly assumed.

The most likely model describing population dynamics of *A. gambiae* s.l. was the rainfall-modified Ricker-logistic density-dependent model that incorporated the mean body size of the maternal and current generation. Density-dependent growth has now been detected in *Culex*, *Aedes* [20,21] and *Anopheles* mosquitoes, and may therefore be a ubiquitously important driver of mosquito vector population dynamics. We observed a negative relationship between population growth and density, indicating that competition becomes an important influence at relatively high population densities. Importantly, the carrying capacity of the population was not fixed and density dependence was evident only after the carrying capacity was adjusted by weekly rainfall. Similarly, density dependence can also be context-dependent for mammals; for example, seasonal resource availability is able to mediate density dependence within large herbivores [40,46], and the carrying capacity of elephant populations is closely linked to annual rainfall [39].

Here we demonstrated that changes in the mean population value of a phenotypic trait, specifically their body size, can affect population-level demographic performance. The rapid expansion of *A. gambiae* s.l. populations observed at the start of the wet season here was probably facilitated by large (and highly fecund) individuals in addition to the increased abundance of larval habitat. After the sharp peak in mosquito density later in the season, the population began to decline as maternal and current wing length fell, despite larval habitat still being highly abundant. This suggests that at this time strong competitive interactions were present in the larval habitats, leading to smaller mosquitoes with reduced reproductive fitness [7]. Of importance is that these forces interact on the larval population—where habitat quality can influence individual fitness [7–9]—and it is unlikely that density-dependent drivers are active among the adult population [47]. Our results demonstrate that the endpoint of this interaction—the individual phenotype—can contribute to population dynamics, but the effect is complex. These results support a growing body of evidence showing that maternal effects are important in driving outbreaks of other insect species [48–50].

The strongest exogenous driver of population dynamics was rainfall, which, by impacting resource availability, is a major driver of population dynamics for a wide range of taxa, from large mammals to insect populations. Regarding *A. gambiae* s.l., the positive association with rainfall has been widely documented [17–19]. It is important to note that models should be tailored to the biological realities of each species—for instance, another mosquito, *Aedes vigilax* (Skuse), was negatively related to rainfall, and the strongest exogenous driver of population dynamics was high-tide frequency [21]. In the current study, the exogenous factors of temperature and RH did not add explanatory value to the model, whereas previous research has demonstrated that mosquito survival is related to these factors [17,19]. The statistical approach adopted in the current study selects for the most representative model using the lowest number of factors [41]. Although the factors of temperature and RH probably influenced survival and demographic rates in the field, the overwhelming influences of rainfall, density dependence and individual phenotype downregulated the importance of these exogenous factors.

The existence of density-dependent feedback has implications regarding the efficacy of techniques used to control the density of this malaria vector. For instance, research on other systems has concluded that perturbations applied to populations with different densities can trigger differing demographic responses [51] and that the timing of control measures, in relation to seasonal fluctuation, may also effect outcomes [4]. Most importantly, low-density populations may have an enhanced ability to compensate for a reduction in numbers by increased reproduction of the remaining individuals. The assumption that endogenous factors play a negligible role on population dynamics could therefore lead to false predictions about the efficacy of density control measures.

The most practical endpoint for vector control programme managers is the elimination of transmission by reducing vector density, and thereby human–vector contact. This endpoint is strongly influenced by environmental stochasticity and density-dependent processes [4,52]. For example, as population densities decrease, the capacity for recovery increases; therefore there is a need to identify strategies that maintain strong larval density dependence even when adult populations are small. One such strategy is to target the availability of larval habitat by larviciding [53] or environmental management [54] rather than solely targeting adult survival (as in conventional insecticide approaches). Strategies that reduce larval resource availability can maintain or even enhance larval competition and its regulatory effects on the adult population. On the other hand, insecticide-based strategies focusing on adults, such as ITNs and indoor residual spraying, would reduce larval density, and therefore could be proportionally less effective at low densities. Future research is required to extend this population dynamic model to incorporate stochastic influences, as well as vector control endpoints and timing of implementation.

There is a need to enrich vector control research with more modern ecological models that can explicitly test for the influence of different population drivers [15]. Sequentially, disease transmission models can be updated to incorporate density-dependent feedback of mosquito populations and ultimately provide a more realistic prediction of adult mosquito survival [55]. Such fundamental baseline information can be used for planning density control measures of this malaria vector, and our results highlight the need to complement traditional methods (e.g. adult-targeted insecticides) with those that will maintain or strengthen natural processes of population regulation (e.g. larviciding or environmental management).

We caution, however, that while such endogenous feedback mechanisms may attenuate the impact of many vector control interventions, this should in no way undermine confidence in, or expenditure upon, such effective malaria control tools as ITNs or indoor residual spraying. These measures are known to cost-effectively prevent malaria in the poorest parts of Africa, saving countless lives as part of ongoing efforts to reduce disease burden on the continent. The low mosquito densities at which the phenomenon outlined here becomes relevant is in itself an indicator of successful control; thus these observations have no implications in terms of whether these imperfect but effective tools should continue to be used at scale. Instead, these observations reinforce the point that these tools alone will not eliminate malaria from most of Africa, and will need to be complemented by strategies that consider such subtleties of mosquito ecology and enable better control of the parasite, vector or both.
